# A Statistical Approach to Optimizing Concrete Mixture Design

**DOI:** 10.1155/2014/561539

**Published:** 2014-02-09

**Authors:** Shamsad Ahmad, Saeid A. Alghamdi

**Affiliations:** Civil and Environmental Engineering Department, King Fahd University of Petroleum and Minerals, Dhahran 31261, Saudi Arabia

## Abstract

A step-by-step statistical approach is proposed to obtain optimum proportioning of concrete mixtures using the data obtained through a statistically planned experimental program. The utility of the proposed approach for optimizing the design of concrete mixture is illustrated considering a typical case in which trial mixtures were considered according to a full factorial experiment design involving three factors and their three levels (3^3^). A total of 27 concrete mixtures with three replicates (81 specimens) were considered by varying the levels of key factors affecting compressive strength of concrete, namely, water/cementitious materials ratio (0.38, 0.43, and 0.48), cementitious materials content (350, 375, and 400 kg/m^3^), and fine/total aggregate ratio (0.35, 0.40, and 0.45). The experimental data were utilized to carry out analysis of variance (ANOVA) and to develop a polynomial regression model for compressive strength in terms of the three design factors considered in this study. The developed statistical model was used to show how optimization of concrete mixtures can be carried out with different possible options.

## 1. Introduction

Optimization of the concrete mixture design is a process of search for a mixture for which the sum of the costs of the ingredients is lowest, yet satisfying the required performance of concrete, such as workability strength and durability. The basic ingredients of concrete can be classified into two groups: cement paste and aggregates. Although the quality of cement paste is governed mainly by the water/cement ratio, the quantity of cement paste required to achieve a targeted quality of concrete depends on the characteristics of aggregates. These characteristics mainly include surface area and voids in aggregates. While surface area is governed by the shape and maximum size of aggregates, the void content is affected mainly by the particle size distribution of aggregates. The requirement of the paste can be reduced by reducing the void content of aggregates through proper packing of the aggregates [1–5] and also by increasing the aggregate/cement ratio [6]. Goltermann et al. [1] have suggested a packing model for the aggregate selection and combination to obtain aggregate mixes having the lowest void contents with maximum packing degree (the ratio between bulk density and the aggregate grain density). Thus, the packing degree according to them is a characteristic of the specific aggregate type or mix and it indicates the void volume and the amount of cement paste necessary in the concrete. This indicates that a concrete mixture design can be optimized by adjusting the levels of the key mixture factors such as water to cementitious materials ratio, coarse aggregate to total aggregate ratio, and cementitious material content or aggregate to cementitious materials ratio as reported by various researchers [7–12].

Attempts have been made in the past to optimize the concrete mixture design using either the fully experimental methods or fully analytical methods or semiexperimental (half-analytical) methods or statistical methods. Fully experimental methods involve an extensive series of tests, sometimes conducted on a trial-and-error basis, and the optimization results are often applicable only to a narrow range of local materials [13, 14]. In order to reduce the number of trial mixtures required to obtain an optimal mixture, efforts have been made towards developing analytical methods rationalizing the initial mixture proportioning into a more logical and systematic process [15]. Analytical methods help in searching for an optimum concrete mixture based on detailed knowledge of specific weights of mixture components and on certain basic formulas, which result from previous experience without conducting expensive and time-consuming experimental works [15, 16]. Semiexperimental (half-analytical) methods are based on combining the experimental database or experimentally developed prediction models and various analytical tools such as artificial neural network, genetic algorithm, and mathematical programming [17–19]. Statistical methods, also termed as statistical experiment design methods or statistical factorial design methods or design of experiments methods or empirical methods, are also used frequently in obtaining the optimum concrete mixture design [9, 10, 20–24]. Statistical methods are an improvement over fully experimental methods, in which, instead of selecting one starting mix proportion and then adjusting by trial and error for achieving the optimum solution, a set of trial batches covering a chosen range of proportions for each mixture component is defined according to established statistical procedures. Trial batches are then carried out, test specimens are fabricated and tested, and experimental results are analyzed using standard statistical methods. These methods include fitting empirical models to the data for each performance criterion. In these models, each response (resultant concrete property) such as strength, slump, or cost is expressed as an algebraic function of factors (individual component proportions) such as w/c, cement content, chemical admixture dosage, and percent pozzolana replacement. After a response can be characterized by an equation (model), several analyses are possible. For example, a user could determine which mixture proportions would yield one or more desired properties. A user also could optimize any property subject to constraints on other properties. Simultaneous optimization to meet several constraints is also possible. For example, one could determine the lowest cost mixture with strength greater than a specified value, air content within a given range, and slump within a given range.

Fully analytical methods are less expensive and less time consuming but they have the disadvantage of being less precise because of the variations in the materials characteristics of the aggregates and cements. Fully experimental or semiexperimental (i.e., half-analytical) methods are reliable and accurate; however, they involve comprehensive laboratory works [16]. Statistical methods also require a certain amount of experimental works but they have an additional advantage in a sense that the expected properties (responses) can be characterized by an uncertainty (variability). This has important implications for specifications and for production of the cost-effective concrete mixture [10].

In the present work, an effort has been made to exhibit the application of a statistical approach proposed to obtain optimum proportioning of concrete mixtures using the data obtained through an experiment design considering water-cementitious materials ratio, cementitious materials content, and fine aggregate to total aggregate ratio as design factors. The experimental data were analyzed statistically and mathematical polynomials regression was developed for concrete strength as a function of mixture variables. The utility of the developed compressive strength model in optimizing the mixture designs was illustrated considering different possible options.

## 2. Proposed Approach

The proposed approach to optimizing the proportions of concrete mixtures is based on the planned experimental works (within the domain of required characteristic performance of concrete) and statistical analysis of the data generated, which would reduce the number of trial batches needed. The proposed approach consists of the following steps.

### 2.1. Specification of the Characteristic Performance of Concrete

The information pertaining to required workability, strength, and exposure conditions (for durability requirements) should be first collected. The workability requirements depend on the mode of transportation, handling, and placing and also on type of construction [25]. The strength is specified based on the structural requirements for concrete protected from exposure to freezing and thawing and application of deicing chemicals or aggressive substances. However, for aggressive exposure conditions, the strength specified by the structural designer should not be less than the minimum design compressive strength recommended for the given exposure condition. For example, ACI 318 [26] has specified minimum design compressive strengths of 28, 31, and 35 MPa, respectively, for concrete exposed to water, freezing-thawing, and chlorides. The durability requirements of concrete mixtures are normally satisfied by ensuring that the cementitious materials content is not less than a specified minimum value and the water/cementitious materials ratio is not more than the one specified for a given exposure condition. For example, the cementitious materials content should not be less than 335 kg/m^3^ and water/cementitious materials ratio should not be more than 0.40 (by mass) for satisfying the durability requirements for concrete subjected to severe exposure conditions such as severe freeze-thaw, deicer, and sulfate exposures [26].

### 2.2. Selection of the Levels of Key Mix Design Factors

Selection of the levels of the three key mixture design factors, namely, cementitious materials content, water/cementitious materials ratio, and fine/total aggregate ratio, which mainly affect the quality of concrete will be made to ensure that enough experimental data are generated for obtaining a regression model for compressive strength which can be used to optimize the mixture proportions meeting the specified characteristic performance of concrete.

The minimum level of cementitious materials content should not be less than 335 kg/m^3^ which is the minimum value to satisfy the durability requirements for aggressive exposure conditions. The maximum level of cementitious materials content should be selected considering the risk of shrinkage. The minimum level of water/cementitious materials ratio should be selected considering the strength requirements. In case of choosing a very low level of the water/cementitious materials ratio, the difficulty in transporting, handling, and placing concrete and extra cost of superplasticizer to meet the workability requirements should be considered. The maximum level of the water/cementitious materials ratio should be within the maximum permissible limit for the water/cementitious materials ratio for the given exposure condition. The minimum and maximum levels of the fine/total aggregate ratio should be selected within the optimum range for achieving maximum packing of aggregates. For example, Soudki et al. [9] have reported optimum fine/total aggregate ratio in the range of 0.40 and 0.45.

### 2.3. Experimental Work for Generating Data to Obtain Statistical Model for Optimization

An experimental work should be conducted involving designing, preparing, and testing various trial mixtures according to the full factorial experiment design considering the various possible combinations of the levels of the mixture variables within their selected ranges of variation. The workability of each trial mixture should be equal to or more than the specified value. In case if superplasticizer is needed to achieve the intended workability, the cost of superplasticizer should be added to the cost of cement. After finalizing the dosage of superplasticizer based on the required workability for each of the trial mixtures, the cubical or cylindrical specimens should be prepared, cured for 28 days, and then tested for compressive strength for generating data to obtain statistical model for strength to be used for optimization.

### 2.4. Statistical Analysis of Experimental Data and Fitting of the Strength Model

Analysis of variance (ANOVA) can be used for examining the significance of the factors considered for developing the strength model and subsequently fitting an empirical model for compressive strength in terms of the significant mixture factors using polynomial regression. In ANOVA, the statistical terminologies used are as follows.

*Degree of Freedom *(*DF*). Degree of freedom is the number of values in the final calculation of a statistic that are free to vary. DF = *n* − 1, where *n* represents the number of groups.

*Error (Residual)*. It is the amount by which an observed variate differs from the value predicted by the assumed statistical model.

*Sum of Squares *(*SS*). It is the squared distance between each data point (*X*_*i*_) and the sample mean ($\bar{X}$), summed for all *n* data points. $\text{SS}={\sum_{i=1}^{n}\left(X_{i}-\bar{X}\right)}^{2}$, where, *X*_*i*_ represents the *i*th observation and $\bar{X}$ represents the sample mean.

*Mean Square *(*MS*). It is the sum of squares divided by the degrees of freedom. 

*F-Ratio.* It is ratio of MS of the concerned factor to the MS of the error. A higher *F-*ratio indicates a significant effect of the factor.

*P-Value. *It is a measure of *acceptance* or *rejection* of a statistical significance of a factor based on a standard that no more than 5% (0.05 level) of the difference is due to chance or sampling error. In other words, if the *P* value for a factor is 0.05 or more, it would not have effect on the dependent variable.

### 2.5. Optimization of Mixture Proportions Using the Fitted Strength Model

The statistical model for the compressive strength derived utilizing the experimental model can be used to obtain the optimal mixture proportions satisfying the specified characteristic performance of concrete as required constraints. The mixture satisfying all the constraints and having the lowest requirements of cement and superplasticizer would be considered as optimum mixture.

## 3. Experimental Program

### 3.1. Test Program

For illustrating the utilization of the proposed approach to optimizing concrete mixture design, an experimental program was considered. A full factorial experiment with 3 × 3 × 3 treatment combinations was used resulting in a total of 27 concrete trial mixtures considering three typical levels of each of the three key factors affecting the performance of concrete mixtures, as shown in Table 1. For the levels of the water/cementitious materials ratio selected in the present work, no superplasticizer was added. The combinations of the levels of the three factors for all 27 trial mixtures are shown in Table 2.

### 3.2. Materials and Mix Proportioning

The cementitious materials used in this study consisted of 92% Type I Portland cement conforming to ASTM C 150 [27] and 8% silica fume (by mass). The crushed stone particles obtained from a local query were used as coarse aggregate and local dune sand was used as fine aggregate. Potable water was used for mixing the constituents of all the specimens. The specific gravity, water absorption, and sieve analysis results for the used coarse and fine aggregates are presented in Table 3. The specific gravities of water and cementitious materials were taken as 1 and 3.15, respectively.

The proportioning of all 27 trial mixtures was carried out in terms of absolute volume using the specific gravities of the concrete ingredients and the values of water/cementitious materials ratio, cementitious materials content, and fine/total aggregate ratio for each of 27 mixtures, as given in Table 2. The water absorption values of fine and coarse aggregates were used to determine the gross water content.

### 3.3. Preparation and Testing of Specimens

Considering three replicates for each of the 27 mixtures, a total number of 81 cylindrical concrete specimens (size: 75 mm diameter and 150 mm high) were cast for determining compressive strength. After casting, the concrete specimens were cured for 28 days in a curing tank under laboratory conditions and then tested for compressive strength in accordance with ASTM C 39 [28]. The average compressive strength of the three specimens made from the same concrete mixture and tested at the same age was considered as characteristic compressive strength of a mixture.

## 4. Results and Discussion

Average 28-day compressive strength test results for all 27 concrete mixtures along with the standard deviation of three replicates of each mixture are presented in Table 4. The data given in Table 4 were utilized for statistical analysis to examine the significance of the mixture factors and subsequently to obtain a regression model for compressive strength in terms of the factors considered.

### 4.1. Statistical Analysis of Data and Fitting of the Compressive Strength Model

Analysis of variance (ANOVA) was carried out to pinpoint the individual and interactive effects of variable factors on the dependent variable. ANOVA of the test results in the present study was done with the software named MINITAB [29]. Based on the ANOVA results, the polynomial regression model for compressive strength was obtained.

The results of ANOVA for compressive strength are presented in Table 5. A factor was considered to have significant effect on the compressive strength if *P* value was found to be less than 0.05 (95% confidence level). The *P* value was obtained from Fisher's distribution table which depends on error degree of freedom (DF) and the mean squares (MS). Table 5 shows that the *R*_w/cm_ and *R*_FA/TA_ have significant effects on compressive strength as their levels of significance, *P* values, are less than 0.05. Therefore, these two significant variables should be considered for obtaining the regression model for compressive strength, *f*_*c*_′. Although the effect of *Q*_*C*_ on compressive strength is found to be insignificant because it varies within a narrow range of 350 to 400 kg/m^3^, it is considered in the regression analysis as cement remains an indispensable material in concrete production.

The polynomial regression model obtained for compressive strength using the data presented in Table 4 is presented as follows:

(1) fc′=−61.24−0.056QC−19.87Exp(2.083Rw/cm)+183.45RFA/TA0.119 (R2=0.80),

where *f*_*c*_′ is the 28-day compressive strength in MPa. *Q*_*C*_ is the cementitious materials content in kg/m^3^. *R*_w/cm_ is the water/cementitious materials ratio by mass. *R*_FA/TA_ is the fine/total aggregate ratio by mass.

The upper and lower bounds of each of the three variables are given in Table 1.

### 4.2. Optimization of Concrete Mixture Proportions Using Compressive Strength Model

The empirical model obtained for compressive strength can be used for optimization of concrete mixture proportions using any suitable optimization method/tool. The developed compressive strength model was utilized for optimization of concrete mixture design corresponding to the following options (i.e., constraints) typically using the *Microsoft Excel Solver*:

1. optimizing the levels of the *R*_w/cm_ and *R*_FA/TA_ for achieving *maximum possible compressive strength* at different values of cementitious materials content within the selected range (i.e., 350, 375, and 400 kg/m^3^),
2. optimizing the levels of the *R*_w/cm_ and *R*_FA/TA_ for achieving different *target compressive strengths* at different values of cementitious materials content within the selected range (i.e., 350, 375, and 400 kg/m^3^).

The optimization results, presented in Table 6, indicate that the maximum compressive strength corresponding to a cementitious materials content of 350 kg/m^3^ is higher than that at cementitious materials contents of 375 and 400 kg/m^3^. Further, the maximum compressive strength at cementitious materials content of 375 kg/m^3^ is higher than that corresponding to a cementitious materials content of 400 kg/m^3^. This indicates that, at the same optimum values of *R*_w/cm_ and *R*_FA/TA_, the compressive strength is more at lower cementitious materials content (i.e., at a higher aggregate to cement ratio) due to better aggregate packing, as reported by Neville and Brooks [6]. At all levels of the cementitious materials content, maximum compressive strengths correspond to minimum water/cementitious materials ratio (0.38) and maximum fine/total aggregate ratio (0.45) within their ranges of variation considered in the present work.

From Table 6, the concrete mixture having a maximum compressive strength of 42.1 MPa at a minimum cementitious materials content of 350 kg/m^3^, water/cementitious materials ratio of 0.38, and fine/total aggregate ratio of 0.45 can be typically selected as the optimum mixture. However, in cases where the compressive strength requirement is less than the maximum, a set of water/cementitious materials ratio, cementitious materials content, and fine/total aggregate ratio other than the optimum one can be selected for achieving the workability and durability requirements.

**Figure 1:**
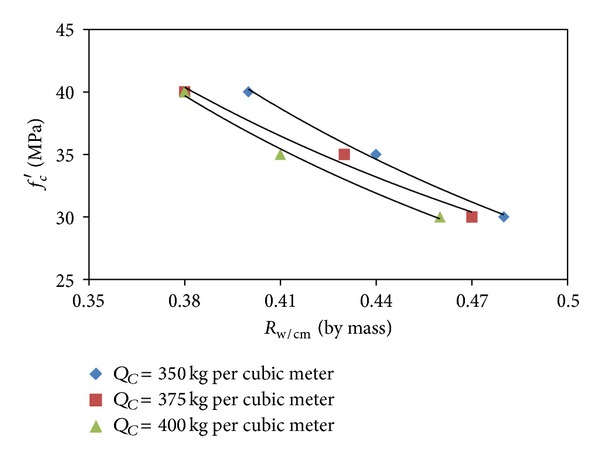
Variation of compressive strength with *R*_w/cm_ at different levels of *Q*_*C*_.

**Figure 2:**
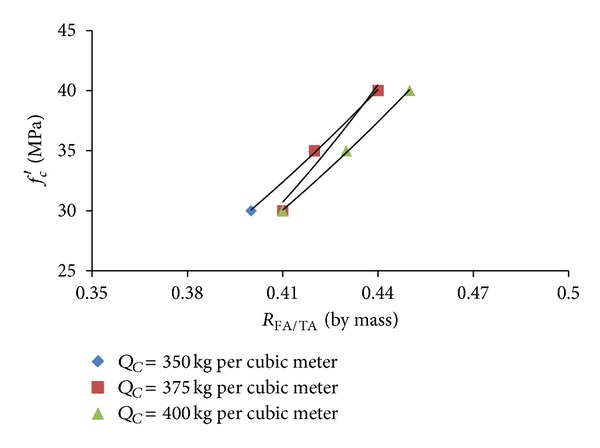
Variation of compressive strength *R*_FA/TA_ at different levels of *Q*_*C*_.

The data obtained from the optimization option (ii), as presented in Table 6, were plotted to depict the variations of compressive strength with water/cementitious materials ratio and fine/total aggregate ratio at different cementitious materials contents, as shown in Figures 1 and 2, respectively. It can be seen from Figures 1 and 2 that, at a given cementitious materials content, the compressive strength increases with the decrease in the water/cementitious materials ratio and the increase in the fine/total aggregate ratio. It can be observed from Figure 1 that, for the same value of compressive strength, the requirement for water/cementitious materials ratio is lower at higher values of the cementitious materials content. Therefore, the plots presented in Figure 1 can be utilized to select an adequate value of the water/cementitious materials ratio and cementitious materials content for a given value of the target compressive strength satisfying the workability and durability requirements. For example, in the case of a normal exposure, a higher value of the water/cementitious materials ratio and a lower value of cementitious materials content can be selected which would give more workability at a lower cost, whereas, for harsh exposure conditions, a lower value of the water/cementitious materials ratio and a higher value cementitious materials content can be selected, which would provide better durability.

## 5. Conclusions

A simplified step-by-step approach is proposed for optimizing the concrete mixture design based on the analysis of the data obtained through a statistically planned experimental program. The proposed approach consists of five steps, as follows: (i) specification of the characteristic performance of concrete, (ii) selection of the levels of key mix design factors, (iii) experimental work considering trial mixtures using full factorial experiment design for generating data to obtain statistical model for optimization, (iv) statistical analysis of experimental data and fitting of the strength model, and (v) optimization of mixture proportions using the fitted strength model.

The results of the experimental works conducted in the present study for demonstrating the utility of the proposed statistical approach have indicated the significant effects of water/cementitious materials ratio, cementitious materials content, and fine/total aggregate ratio on compressive strength. The optimum values of water/cementitious materials ratio and fine/total aggregate ratios have resulted in a higher compressive strength at a lower cementitious materials content resulting in significant cost saving in the concrete production.

## Figures and Tables

**Table 1:** Test program.

Factor	1	2	3	Level
Cementitious materials content (*Q*_*C*_) in kg/m^3^	350	375	400	3

Water/cementitious materials ratio (*R*_w/cm_) by mass	0.38	0.43	0.48	3

Fine/total aggregate ratio (*R*_FA/TA_) by mass	0.35	0.40	0.45	3

**Table 2:** Trial mixtures.

Mix number	Water/cementitious materials ratio (*R*_w/cm_)	Cementitious materials content, *Q*_*C*_ (kg/m^3^)	Fine/total aggregate ratio (*R*_FA/TA_)
1	0.38	350	0.35
2	0.38	350	0.40
3	0.38	350	0.45
4	0.38	375	0.35
5	0.38	375	0.40
6	0.38	375	0.45
7	0.38	400	0.35
8	0.38	400	0.40
9	0.38	400	0.45
10	0.43	350	0.35
11	0.43	350	0.40
12	0.43	350	0.45
13	0.43	375	0.35
14	0.43	375	0.40
15	0.43	375	0.45
16	0.43	400	0.35
17	0.43	400	0.40
18	0.43	400	0.45
19	0.48	350	0.35
20	0.48	350	0.40
21	0.48	350	0.45
22	0.48	375	0.35
23	0.48	375	0.40
24	0.48	375	0.45
25	0.48	400	0.35
26	0.48	400	0.40
27	0.48	400	0.45

**Table 3:** Specific gravity, water absorption, and sieve analysis of aggregates.

Sieve size	Cumulative percentage retained
(a) Coarse aggregate (specific gravity = 2.55; water absorption = 1.1%)	

19 mm	5
12.5 mm	60
9.5 mm	95
4.75 mm	100

(b) Fine aggregate (specific gravity = 2.66; water absorption = 0.6%)	

1.18 mm	0
0.60 mm	24.42
0.30 mm	90.49
0.15 mm	96.59

**Table 4:** Compressive strength test results.

Mix number	28-day average compressive strength, *f*_*c*_′ (MPa)	Standard deviation of three replicates of each mixture (MPa)
1	39.7	1.9
2	38.8	1.0
3	39.1	0.8
4	34.1	1.2
5	38.2	1.9
6	40.6	2.0
7	34.2	1.1
8	39.3	1.1
9	39.8	1.6
10	27.9	1.1
11	37.4	1.8
12	38.5	1.1
13	31.9	0.8
14	37.1	1.3
15	33.9	0.2
16	26.5	1.4
17	30.7	1.7
18	36.5	1.6
19	30.0	1.5
20	32.1	1.3
21	30.5	0.8
22	20.7	1.8
23	27.5	0.8
24	29.9	0.3
25	25.4	1.1
26	31.0	0.2
27	25.3	0.2

**Table 5:** ANOVA for compressive strength test results.

Factors	Type	Level	Scale values			
*Q*_*C*_	Fixed	3	0.875	0.938	1.000	
*R*_w/cm_	Fixed	3	0.792	0.896	1.000	
*R*_FA/TA_	Fixed	3	0.778	0.889	1.000	

Source	DF	SS	MS	*F*-ratio	*P* value	Significance

*Q*_*C*_	2	39.672	19.380	1.990	0.199	No
*R*_w/cm_	2	464.501	232.250	23.260	0.000	Yes
*R*_FA/TA_	2	135.281	67.640	6.770	0.019	Yes
*Q*_*C*_ ∗ *R*_w/cm_	4	23.686	5.921	0.590	0.678	No
*Q*_*C*_ ∗ *R*_FA/TA_	4	4.993	1.248	0.120	0.969	No
*R*_w/cm_ ∗ *R*_FA/TA_	4	22.437	5.609	0.560	0.697	No
Error	8	79.890	9.986			

Total	26	770.456				

**Table 6:** Optimization of concrete mixture design.

Optimization option	*f*_*c*_′ (MPa)	Optimum levels of the mixture variables			Cost level
		*Q*_*C*_ (kg/m^3^)	*R*_w/cm_ (by mass)	*R*_FA/TA_ (by mass)	
(i) Optimizing the levels of *R*_w/cm_ and *R*_FA/TA_ for achieving *maximum compressive strengths* at different levels of *Q*_*C*_	42.1	350	0.38	0.45	Low
	40.7	375	0.38	0.45	Medium
	39.3	400	0.38	0.45	High

(ii) Optimizing the levels of *R*_w/cm_ and *R*_FA/TA_ for achieving different *target compressive strengths* at different levels of *Q*_*C*_	30.0		0.48	0.40	
	35.0	350	0.44	0.42	Low
	40.0		0.40	0.44	
	30.0		0.47	0.41	
	35.0	375	0.43	0.42	Medium
	40.0		0.38	0.44	
	30.0		0.46	0.41	
	35.0	400	0.41	0.43	High
	40.0		0.38	0.45	
